# Loss of p300 and CBP disrupts histone acetylation at the mouse *Sry* promoter and causes XY gonadal sex reversal

**DOI:** 10.1093/hmg/ddx398

**Published:** 2017-11-14

**Authors:** Gwenn-Aël Carré, Pam Siggers, Marilena Xipolita, Paul Brindle, Beat Lutz, Sara Wells, Andy Greenfield

**Affiliations:** 1 Mammalian Genetics Unit, Medical Research Council, Harwell Institute, Oxfordshire OX11 0RD, UK; 2 Department of Biochemistry, St. Jude Children’s Research Hospital, Memphis, TN 38105, USA; 3 Institute of Physiological Chemistry, University Medical Center Mainz, 55128 Mainz, Germany; 4 Mary Lyon Centre, Medical Research Council, Harwell Institute, Oxfordshire OX11 0RD, UK

## Abstract

CREB-binding protein (CBP, CREBBP, KAT3A) and its closely related paralogue p300 (EP300, KAT3B), together termed p300/CBP, are histone/lysine acetyl-transferases that control gene expression by modifying chromatin-associated proteins. Here, we report roles for both of these chromatin-modifying enzymes in mouse sex determination, the process by which the embryonic gonad develops into a testis or an ovary. By targeting gene ablation to embryonic gonadal somatic cells using an inducible Cre line, we show that gonads lacking either gene exhibit major abnormalities of XY gonad development at 14.5 dpc, including partial sex reversal. Embryos lacking three out of four functional copies of *p300/Cbp* exhibit complete XY gonadal sex reversal and have greatly reduced expression of the key testis-determining genes *Sry* and *Sox9*. An analysis of histone acetylation at the *Sry* promoter in mutant gonads at 11.5 dpc shows a reduction in levels of the positive histone mark H3K27Ac. Our data suggest a role for CBP/p300 in testis determination mediated by control of histone acetylation at the *Sry* locus and reveal a novel element in the epigenetic control of *Sry* and mammalian sex determination. They also suggest possible novel causes of human disorders of sex development (DSD).

## Introduction

In mammals, the development of the bipotential embryonic gonad as a testis or ovary is determined by the presence or absence of the Y chromosome. In the mouse, the gonads begin to form on the surface of the mesonephros by 9.5 days *post coitum* (dpc) with a thickening of the coelomic epithelium. In XY embryos, the transient expression of *Sry* (sex determination region on Y chromosome) in somatic cells of the undifferentiated gonad between 10.5 dpc and 12.0 dpc triggers testis differentiation (1). SRY, in conjunction with NR5A1 (SF1), directly upregulates expression of *Sox9* (*Sry*-related HMG box 9) (2) and SOX9 activates a gene regulatory network that controls the differentiation of Sertoli cells and morphogenetic events required for testis formation, including formation of testis cords [reviewed in (3)]. In XX gonads, the absence of SRY results in the activation of ovary-promoting pathways involving RSPO1/WNT4/CTNNB1 and FOXL2 (4–9).

The timing of the expression of *Sry* is crucial for activation of the testis-determining pathway of gene expression; a developmental window of approximately 6 h exists in which *Sry* expression is critical (10). Several transcription factors, such as WT1 (+KTS), NR5A1, GATA4/FOG2 and SIX1/4, in addition to the insulin receptor (INSR/IGF1R) and GADD45γ/MAP3K4/p38 MAPK signalling pathways have been shown to be required for a normal *Sry* expression profile (11–18). More recently, a histone lysine demethylase, JMJD1A (KDM3A), has been shown to modulate *Sry* expression through the regulation of H3K9me2 marks at its promoter (19).

The cyclic AMP response element-binding protein (CREB)-binding protein, CBP (also known as CREBBP or KAT3A), and p300 (EP300 or KAT3B) are two members of the KAT3 subfamily of histone acetyl-transferases (HATs). In addition to their ability to acetylate histones and transcription factors, they regulate gene transcription by recruiting transcription factors or by connecting them to the basal transcription machinery [reviewed in (20)]. CBP and p300 are generally associated with promoting active transcription but, depending on the context, they can also act as repressors (21,22). CBP and p300 are implicated in a range of biological processes, including cell differentiation, cell proliferation, tumourigenesis and apoptosis. Embryos lacking either functional p300 or CBP die early due to multiple abnormalities. Loss of p300 disrupts embryonic organogenesis, causing heart and neural tube defects (23). CBP-deficient embryos exhibit neural tube defects, abnormal blood vessels and haemorrhage, which causes death around mid-gestation (23,24). These phenotypes are consistent with clinical features of Rubinstein-Taybi syndrome, which include mental retardation, heart disease and cataracts. *CBP* is commonly mutated in patients with this syndrome, *p300* less frequently (25). Conditional gene targeting has revealed important roles for these molecules in different contexts, with redundant and non-redundant components. Such roles include T-cell development (26), photoreceptors (27), lens induction (28), spermatogenesis (29) and neural functions (30).

Both *p300* and *Cbp* have been shown to be expressed in different cell lineages of the embryonic gonad in mice. Whilst there are no reports of *in vivo* roles for CBP/p300 in sex determination, a number of lines of evidence suggest that further investigation of this possibility is warranted, especially in the context of the function of SRY and SOX9: i) p300/CBP activity is positively implicated in functionality of the testis-determining gene *SOX9* in other contexts, either by promoting *SOX9* transcription (31), or by recruitment to the SOX9 transcriptional machinery itself (32–34); ii) p300/CBP interact with CITED2, a known regulator of *Sry* expression (35,36); iii) the putative human gonadal *SOX9* enhancer element, *RevSex*, contains several p300 binding-sites (37–39); iv) p300-mediated acetylation of human SRY promotes its nuclear localisation (40).

These data suggest that CBP and p300 might play important roles in testis determination or differentiation. However, testing this hypothesis has been precluded by the death, at around mid-gestation, of embryos lacking these genes (23,24). Here, using an inducible Cre/LoxP system, we describe deletion of *p300* and/or *Cbp* in gonadal somatic cells prior to sex determination. Loss of either molecule individually results in partial XY gonadal sex reversal; loss of three out of four functional alleles, retaining either one functional *p300* or one *Cbp* allele, causes complete XY gonadal sex reversal. Careful analysis of mutant gonads reveals that this sex reversal is associated with a reduction in expression of *Sry* and *Sox9* at 11.5 dpc. Chromatin immunoprecipitation reveals a correlative reduction in levels of the positive histone acetylation mark H3K27Ac at the *Sry* promoter in mutant gonads at the same stage. Our data suggest a requirement for these HATs in establishing a normal profile of *Sry* expression mediated by histone acetylation at its promoter. They also indicate that *CBP* or *p300* sequence variants identified in humans with disorders of sex development (DSD) warrant further investigation in respect of their possible pathogenic effects.

## Results

### *Wt1^ERT2Cre^*or tamoxifen treatment does not significantly disrupt testis determination

To study the role of CBP and p300 in sex determination, conditional deletion in embryonic gonadal somatic cells was performed by crossing *Cbp^flox/flox^*or *p300^flox/flox^*mice with mice carrying *Cbp^flox^*or *p300^flox^* and tamoxifen-inducible *Wt1CreERT2*, in which Cre expression is driven by the endogenous *Wt1* regulatory elements (41). *Wt1* is first expressed in the coelomic epithelium of the urogenital ridge as early as 9.0 dpc (42) and expression continues in somatic cells of both the developing XY and XX gonad (43,44). Gene deletion was induced by tamoxifen administration at 9.5 dpc, prior to sex determination. To assess the efficiency of deletion, we analysed CBP and p300 protein levels in gonads at 14.5 and 11.5 dpc using immunohistochemistry (Supplementary Material, Fig. S1A and B). CBP and p300 were essentially ubiquitous in XY control (*Wt1^ERT2Cre/+^*) gonads and the adjacent mesonephros at both stages, while gonads predicted to lack CBP [*Wt1^ERT2Cre/+^*, *Cbp^flox/flox^* (henceforth, *Cbp* cKO)] or p300 [*Wt1^ERT2Cre/+^*, *p300^flox/flox^* (*p300* cKO)] exhibited a strong reduction in CBP and p300 protein levels, respectively. These data suggest that one dose of tamoxifen at 9.5 dpc is sufficient to efficiently delete these two genes in the somatic cells as early as 11.5 dpc. However, as *Wt1* is not expressed in germ cells, CBP and p300 were detected in these cells (Supplementary Material, Fig. S1A and B).

The *Wt1^ERT2Cre^*line is a knock-in and therefore embryos carrying this Cre are heterozygous for a *Wt1* null allele. WT1 functions in the establishment of the early bipotential gonad and the up-regulation of the testis-determining gene *Sry* (15,45). In order to analyse the effect of *Wt1* heterozygosity on sex determination, we performed wholemount *in situ* hybridization (WIMSH) for *Sox9*, a major gene controlling testis differentiation and a Sertoli cell marker, on gonads from *Wt1^ERT2Cre/+^* embryos, with and without tamoxifen dosing of the dam (Supplementary Material, Fig. S2A and B). Testis development and *Sox9* expression at 14.5 dpc were not significantly affected by the presence of *Wt1^ERT2Cre^* or affected by tamoxifen treatment. However, a few cells positive for *Stra8*, a gene expressed in pre-meiotic germ cells, usually only detected in XX gonads at this stage, were observed at the poles of one XY gonad carrying *Wt1^ERT2Cre^*and treated with tamoxifen (Supplementary Material, Fig. S2B). This phenotype was also occasionally observed in XY gonads carrying *Wt1^ERT2Cre^*in the absence of tamoxifen treatment (data not shown). These results suggest that XY *Wt1^ERT2Cre/+^*gonads treated with tamoxifen can occasionally exhibit a short delay in testis determination, which explains the polar *Stra8* expression; this is the case with other mutants that have a very minor effect on testis determination (46). No effect of *Wt1^ERT2Cre^*or tamoxifen was observed on XX gonad differentiation (data not shown). It is likely that these gonads are sensitized to any further disruptions to testis determination, rather like the situation in which B6 mice carry the *domesticus* Y chromosome from the AKR strain (B6.Y^AKR^) (47,48). Therefore, for the rest of the study, we used gonads carrying *Wt1^ERT2Cre^*treated with tamoxifen as controls.

### XY gonads following conditional deletion of *Cbp* or *p300* exhibit partial sex reversal

**Figure 1.**
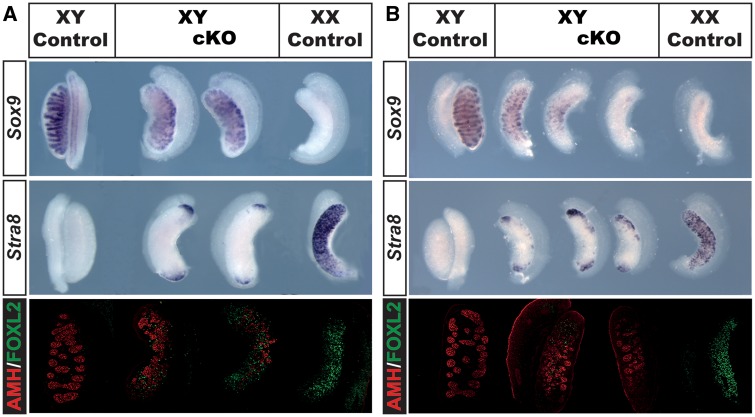
Partial sex reversal in XY *Cbp* cKO and *p300* cKO gonads. Analysis of XY gonads following targeted ablation of *Cbp* [*Wt1^ERT2Cre/+^*, *Cbp^flox/flox^* (*Cbp* cKO)] (**A**) or *p300* [*Wt1^ERT2Cre/+^*, *p300^flox/flox^* (*p300* cKO)] (**B**) reveals partial gonadal sex reversal characterised by disruption to the expression of Sertoli cell markers *Sox9*, analysed by wholemount *in situ* hybridisation (WMISH) (top panel), and anti-Müllerian hormone (AMH), analysed by immunohistochemistry (bottom panel, AMH immunostaining in red). XY mutant gonads express the ovarian germ cell marker *Stra8* (middle panels) at the poles as well as granulosa cell marker FOXL2 to variable degrees (bottom panels, green signal in immunostaining).

To determine the effect of induced conditional deletion of *Cbp* and *p300* on gonad differentiation, we analysed gonads at 14.5 dpc, a stage at which disruptions to sex determination are easily detectable. As expected, XY control gonads (*Wt1^ERT2Cre/+^*) exhibited well differentiated testis cords expressing *Sox9* (Fig. 1A and B, left), while in XX gonads typical ovarian morphology accompanied by strong expression of *Stra8* was observed (Fig. 1A and B, right). XY gonads lacking *Cbp* (*Cbp* cKO) or *p300* (*p300* cKO) (Fig. 1A and B, respectively) exhibited a partial male-to-female gonadal sex reversal phenotype. The overall shape of the mutant gonads was curved, similar to XX control gonads. XY *Cbp* cKO gonads expressed *Sox9* and AMH, markers of Sertoli cells, but testis cord organization was irregular (Fig. 1A). Furthermore, XY *Cbp* cKO gonads expressed *Stra8* at the poles, a phenotype generally associated with a delay in the activation of the male pathway at the centre of the gonad (12,48,49). Immunohistochemistal analyses of FOXL2, normally detected in granulosa cells of XX gonads at this stage, revealed some positive cells in the XY *Cbp* cKO gonads, indicating that some XY supporting cells had adopted an ovarian fate. A similar phenotype of partial gonadal sex reversal was observed in XY *p300* cKO embryos at the same stage (Fig. 1B). However, there was more variability, as illustrated by *Sox9* WIMSH; *Sox9* was absent in some XY *p300* cKO gonads, suggesting nearly complete sex reversal, while some expressed *Sox9* and AMH in association with malformed testis cords. Moreover, *Stra8* expression at the poles of the XY *p300* cKO gonads extended further than in the XY *Cbp* cKO gonads, suggesting a more severe phenotype in the absence of p300 than in the absence of CBP. FOXL2 was also detected in XY *p300* cKO gonads (Fig. 1B).

### XY gonads lacking two copies of *p300* and one copy of *Cbp* exhibit complete sex reversal

**Figure 2.**
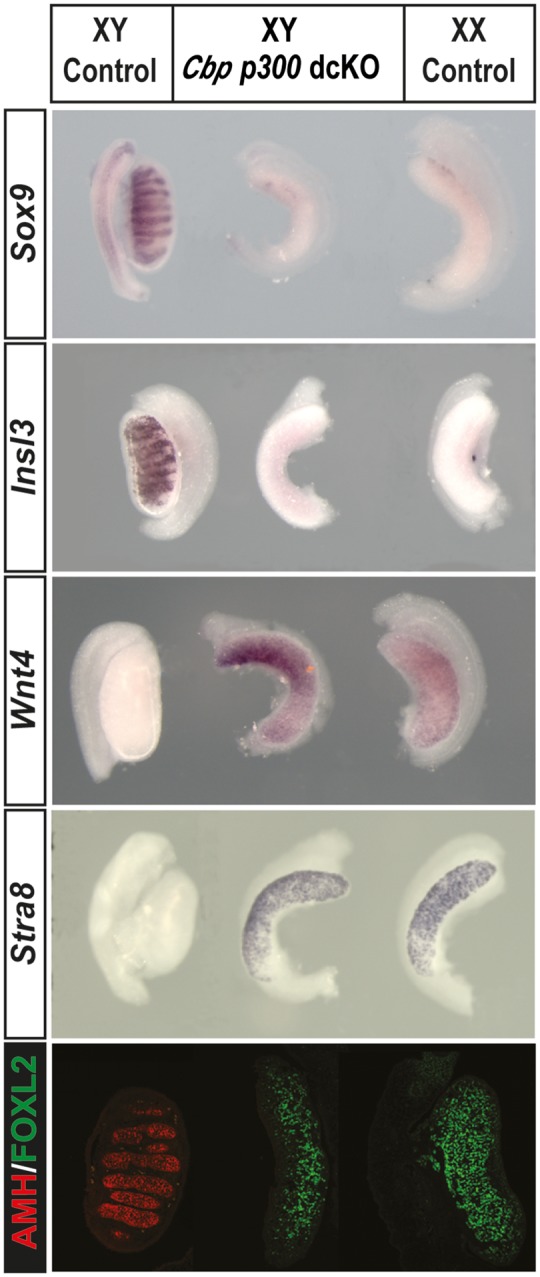
Complete gonadal sex reversal in XY *Cbp/p300* dcKO gonads. XY *Cbp/p300* dcKO (*Wt1^ERT2Cre/+^, Cbp^flox/+^, p300^flox/flox^*) gonads at 14.5 dpc exhibit an ovarian morphology, with loss of *Sox9*, Leydig cell marker *Insl3* (WIMSH) and AMH (immunostaining, red signal). Sex reversal is confirmed by ectopic expression of granulosa cell marker *Wnt4*, meiotic germ cell marker *Stra8* (WMISH) and granulosa cell marker FOXL2 (immunostaining, green signal).

In other contexts, it has been shown that *p300* and *Cbp* can act redundantly (23). Therefore, in order to address whether *Cbp* can compensate for the loss of *p300*, we generated XY gonads conditionally lacking two copies of *p300* and one copy of *Cbp* [*Wt1^ERT2Cre/+^, Cbp^flox/+^, p300^flox/flox^* (*Cbp/p300* dcKO)] (Fig. 2). *Cbp/p300* dcKO XY gonads exhibited complete sex reversal at 14.5 dpc, as evidenced by gonads with an ovarian morphology that lacked expression of Sertoli cell markers (*Sox9*, AMH) or a Leydig cell marker (*Insl3*), but which showed ectopic expression of female somatic and germ cell markers (*Wnt4* or FOXL2, *Stra8*), similar to XX control gonads. XX *Cbp, p300* dcKO gonads showed no overt abnormalities (data not shown). These data show that all testicular cell lineages examined have been affected in *Cbp/p300* dcKO gonads, consistent with wholesale XY ovary development. In a similar fashion, XY embryos with conditional deletion of two copies of *Cbp* and one copy of *p300* [*Wt1^ERT2Cre/+^, Cbp^flox/flox^, p300^flox/+^* (*p300/Cbp* dcKO)] also exhibited complete gonadal sex reversal (Supplementary Material, Fig. S3A). XY gonads lacking one copy of each gene [*Wt1^ERT2Cre/+^, Cbp^flox/+^, p300^flox/+^* (*Cbp/p300* dcHet)] exhibited an intermediate phenotype with disruption of testis cord organization and significant expression of *Stra8* and FOXL2 at the gonadal poles (Supplementary Material, Fig. S3B). These data suggest that CBP and p300 both play a role during testis determination and one can at least partially compensate for loss of the other.

### Reduced *Sry and Sox9* expression in XY *Cbp*/*p300* dcKO gonads at 11.5 dpc

**Figure 3.**
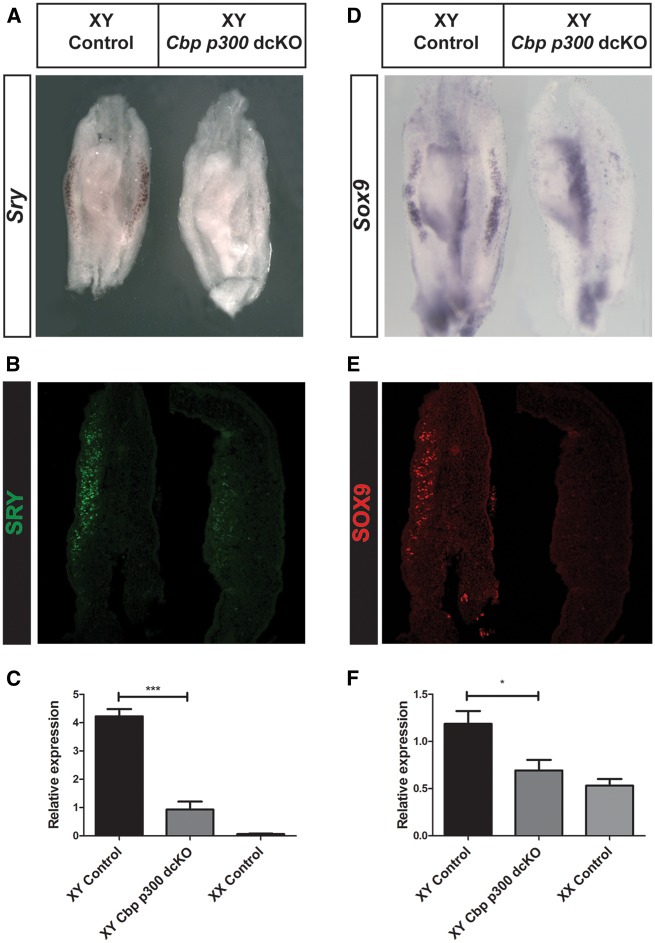
Loss of *Sry* and *Sox9* expression in XY *Cbp/p300* dcKO gonads at the sex-determining stage of 11.5 dpc. Analysis by WIMSH of *Sry* (**A**) and *Sox9* (**D**) showing strong expression in XY control gonads (*Wt1^ERT2Cre/+^*, left gonad) but not in XY *Cbp/p300* dcKO (*Wt1^ERT2Cre/+^*, *Cbp^flox/+^*, *p300^flox/flox^*, right gonad). Anti-SRY (**B**) and anti-SOX9 (**E**) immunostaining detects proteins in XY control gonad (left) but strong reduction in XY *Cbp/p300* dcKO (right gonad). Analysis by qRT-PCR of *Sry* (**C**) and *Sox9* (**E**) mRNA levels confirms data generated by WIMSH and immunohistochemistry. qPCR data are presented as mean ± s.e.m (*n* = 6). *P ≤ 0.05; *** *P ≤ *0.001 (calculated using two-tailed Student’s *t*-test).

In order to determine the molecular basis of the observed gonadal sex reversal at 14.5 dpc, we analysed the expression of *Sry* and its principal target gene, *Sox9*, at 11.5 dpc, at the time testis determination is initiated. *Sry* mRNA and SRY protein levels were drastically reduced in *Cbp*/*p300* dcKO gonads compared with XY controls (Fig. 3A and B). Quantitative analysis of the level of *Sry* in sub-dissected gonads (lacking the mesonephros) by qRT-PCR revealed a reduction of approximately 6-fold in *Cbp*/*p300* dcKO gonads (Fig. 3C). Analysis of *Sox9* by WIMSH (Fig. 3D) and immunohistochemistry (Fig. 3E) revealed an absence of positive cells in *Cbp*/*p300* dcKO gonads, consistent with the phenotype observed at 14.5 dpc. The reduction in levels of *Sox9* transcript, to approximately those found in XX controls, was confirmed by qRT-PCR (Fig. 3F). These data suggest that a failure to attain normal threshold levels of *Sry* at 11.5 dpc, with a consequent loss of *Sox9*, underlies gonadal sex reversal in *Cbp*/*p300* dcKO gonads.

### Disrupted histone acetylation at the *Sry* promoter in XY *Cbp*/*p300* dcKO gonads

**Figure 4.**
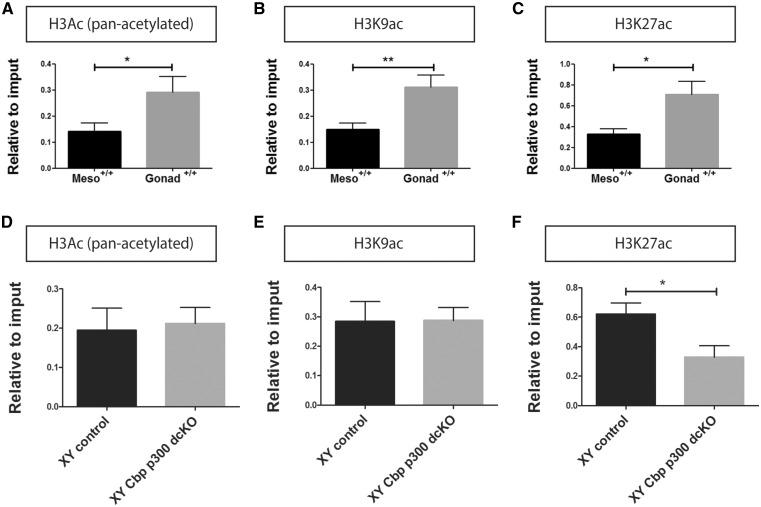
Levels of histone acetylation at the *Sry* promoter at 11.5 dpc. Chromatin immunoprecipitation (ChIP) analyses for H3ac (pan-acetylated H3) (**A**), H3K9ac (**B**) and H3K27ac (**C**) at the *Sry* promoter reveals an enrichment of histone acetylation in XY control gonads compared with mesonephros, which lacks *Sry* expression. Loss of *p300* and C*bp* [*Cbp/p300* dcKO (*Wt1^ERT2Cre/+^*, *Cbp^flox/+^*, *p300^flox/flox^)*] results in a significant decrease in the levels of H3K27ac (**F**) at the *Sry* promoter in comparison to controls, whilst levels of H3ac (pan-acetylated H3) (**D**) and H3K9ac (**E**) were unaffected. All data are presented as mean ± s.e.m. (*n* ≥ 6). *P ≤ 0.05 (Student’s *t*-test).

Our data reveal a role for CBP/p300 in testis determination via a positive impact on *Sry* expression. Finally, we investigated a potential link between these two factors and activating histone marks at the *Sry* promoter using chromatin-immunoprecipitation (ChIP) from whole gonads (lacking the attached mesonephros) at 11.5 dpc (Fig. 4; see Materials and Methods for details). We determined the level of histone H3 acetylation of all lysine residues (pan-acetylated H3), lysine 9 (H3K9ac) and 27 (H3K27ac). K27 is a preferential target of CBP/p300 acetylation, whilst K9 is acetylated by other histone acetyl-transferases, such GCN5/PCAF (50). When compared with the mesonephros, these three marks were enriched at the *Sry* promoter in XY B6 wild-type gonads, suggesting a correlation between histone H3 acetylation and *Sry* expression (Fig. 4A–C). Crucially, there was a reduction in the level of H3K27ac at the *Sry* promoter of *Cbp/p300* dcKO gonads when compared with XY controls (tamoxifen-treated, *Wt1^ERT2Cre/+^*) (Fig. 4F). In contrast, the levels of pan-acetylated H3 and H3K9ac remained unchanged in *Cbp/p300* dcKO gonads compared with XY controls (Fig. 4D and E). These data suggest that CBP/p300 activity is required for the establishment of a normal profile of positive histone acetylation marks at the *Sry* promoter.

## Discussion

Here we demonstrate for the first time a role for CBP/p300 in mammalian testis determination. Using an inducible Cre recombinase system to circumvent the embryonic lethality seen in *Cbp* and *p300* knockout mice, we observed a partial male-to-female gonadal sex reversal in XY embryos with targeted ablation of either *Cbp* or *p300* in somatic cells. Complete gonadal sex reversal occurred when only three out of four functional copies of *Cbp/p300* were targeted for deletion. However, it should be remembered that the presence of *Wt1^ERT2Cre^* means that embryos are haploinsufficient for *Wt1*, a gene with a known role in testis determination. Although tamoxifen-treated *Wt1^ERT2Cre^* XY embryos develop testes as normal, it is likely that this haploinsufficiency makes some small contribution to the sex reversal phenotype. It is possible that in the presence of two wild-type copies of *Wt1*, it would be necessary to delete all four functional copies of *Cbp/p300* in order to effect XY gonadal sex reversal.

Our observation that partial gonadal sex reversal is caused by deletion of either gene indicates that both CBP and p300 play a role during testis determination. Although we observed a strong decrease of CBP and p300 proteins in the somatic cells of the gonads at 11.5 dpc, we cannot formally exclude the possibility that deletion does not occur in all cells, or that this deletion is not early enough, and that, as a consequence, some residual p300 or CBP activity is responsible for the observed partial induction of testis differentiation in single gene mutants. However, previous studies have suggested that CBP and p300 can act redundantly. Both compound mutants exhibited complete gonadal sex reversal, with XY gonads developing as ovaries. These data suggest that both genes are involved in testis determination and can at least partially compensate for each other’s loss. Finally, it is noteworthy that sex-reversed mutant XY gonads robustly express *Stra8* and FOXL2, suggesting that the ovarian program of differentiation, in contrast to the testicular, can occur when there is a major functional deficit in these two proteins.

We have demonstrated that, in the *p300/Cbp* dcKO, *Sry* expression is dramatically decreased at 11.5 dpc. This is sufficient to explain the loss of *Sox9* expression at the same stage (2), and account for the sex reversal observed at 14.5 dpc. We sought a mechanistic explanation for this loss of *Sry* expression. Lysine 27 of histone H3 (H3K27), a known target of CBP/p300 acetylation, shows higher levels of acetylation at the *Sry* promoter in XY gonads than in the mesonephros at the time of sex determination. Levels of pan-acetylated H3 and acetylated H3K9 are also higher in the gonad, suggesting a positive correlation between the level of H3 acetylation and *Sry* expression. These experiments were performed on whole gonads and, due to the heterogeneous collection of cell-types found in the gonad at this stage, may under-estimate the enrichment of acetylated H3 at the *Sry* promoter in gonadal somatic cells. In gonads depleted for both CBP and p300 using our conditional deletion strategy, the levels of H3K27 acetylation (H3K27ac) were significantly decreased, suggesting that the down-regulation of *Sry* in the absence of sufficient *Cbp/p300* activity is caused by reduced histone acetylation at its promoter. Our data shed new light on a key developmental gene and its epigenetic control, which remains poorly understood (51). This is only the second report of an altered epigenetic mark at the *Sry* promoter in a sex-reversed mutant. Elevated levels of H3K9me2, an inhibitory mark, were observed in embryonic gonads lacking the H3K9 histone demethylase, JMJD1A (19). These data, taken together with those reported here, reveal a complexity of epigenetic regulation at the *Sry* locus required for its normal expression and, as a consequence, testis determination.

Levels of H3K9ac are higher in wild-type gonads than in mesonephros, but are unaffected by the loss of *Cbp/p300*, suggesting that some other unidentified histone acetyl-transferases play a role in chromatin modification at the *Sry* locus. Notwithstanding the impact of CBP/p300 activity on chromatin at the *Sry* promoter, which is required to initiate testis determination, we cannot exclude an indirect role i.e. functionally upstream of *Sry*, perhaps via the regulation of expression of transcription factors involved in the regulation of *Sry* itself; however, qRT-PCR analysis of some known regulators of *Sry* (*Wt1*, *Cited2*, *Cbx2*, *Gata4* and others) revealed no differences between CBP/p300-deficient gonads and controls (Supplementary Material, Fig. S4). It should also be noted that in addition to their role as histone acetyl-transferases, CBP and p300 can also acetylate and regulate the activity of non-histone proteins, acting as gene regulatory hubs with at least 400 identified interacting partners, among them known regulators of *Sry* such as WT1, SF1, FOG2, GATA4 and (52).

Finally, a number of recent studies describe the use of exome sequencing of human patients with disorders of sex development (DSD), including partial and complete 46, XY gonadal dysgenesis (sex reversal), as a way of identifying the mutations causally responsible for the observed condition. It is noteworthy that two such studies report potentially disruptive mutations in human *CBP* and *p300* (53,54). Whilst the conditional gene targeting strategy described here does not directly model the effects of constitutively acting human *CBP/p300* gene mutations, it suggests that such variants may act by disrupting cell type-specific events, such as protein-protein interactions, required for human sex determination. The data reported here concerning the requirement for CBP and p300 in mouse testis determination suggest that such variants should be considered as potentially contributing to defects in human testis determination and warrant further investigation.

## Materials and Methods

### Mouse lines and generation of embryos

Mouse experimentation was approved by the Animal Welfare and Ethical Review Body at MRC Harwell. Breeding was performed by license under the Animals (Scientific Procedures) Act with approval from the U.K. Home Office (PPL 70/8898). Mice were housed in individually ventilated cages in a specific pathogen-free environment. Further details of micro- and macro-environmental conditions are available on request. Adult mice were humanely euthanized by dislocation of the neck and embryos were decapitated in ice-cold, phosphate-buffered saline solution.

The *Cbp^flox^* and *p300^flox^* lines have been previously described (55,56) and were maintained on the C57BL/6J (B6) background. In order to target gene deletion to somatic cells of the embryonic gonad, floxed mice were crossed with mice carrying the tamoxifen-inducible *Wt1^ERT2Cre^*allele (41), also maintained on B6. Different crosses were performed to generate mutant tissue for this study: i) *Wt1^ERT2Cre/+^, Cbp^flox/+^*x *Cbp^flox/flox^*; ii) *Wt1^ERT2Cre/+^*, *p300^flox/+^*x *p300^flox/flox^*; or iii) *Wt1^ERT2Cre/+^*, *p300^flox/+^*, *Cbp^flox/flox^* x *p300^flox/flox^*. Deletion was induced with a single dose of tamoxifen (Sigma T5648), dissolved in corn oil, at 200mg/kg per mouse, administered by oral gavage at 9.5 days *post coitum* (dpc). Noon on the day of the copulatory plug was counted as 0.5 dpc.

### Quantitative RT-PCR

Total RNA was extracted from one pair of gonads, dissected away from the mesonephroi, using the RNeasy plus microkit (Qiagen). 300 ng of RNA was reverse-transcribed using the high capacity RNA to cDNA kit (Applied Biosystem). qPCR of cDNA (qRT-PCR) was performed on a 7500 Real-Time PCR system (Applied Biosystems) using Fast SYBR Green Master Mix (Applied Biosystems) according to manufacturer’s instructions At least 3 samples for each genotype were analysed. Data were normalized to *Hprt1*. Fold-change in expression was determined by the 2^−ΔΔCT^ method. Statistical differences were determined by using a two-tailed Student’s *t*-test. Primers sequences are available on request.

### Immunohistochemistry

Tissues were fixed overnight in 4% paraformaldehyde, embedded in paraffin and cut into 8μm sections. Analyses were performed on at least two independent samples per experiment. The following primary antibodies were used: AMH (SC28912, Santa-Cruz), SOX9 (AB5535, Millipore), FOXL2 (a kind gift from Dagmar Wilhelm), SRY (a kind gift from Makoto Tachibana), CBP (SC583, Santa-Cruz) and p300 (SC585, Santa-Cruz). Secondary antibodies were Alexafluor 594 and Alexafluor 488. Images were captured using a Zeiss 710 multiphoton microscope.

### Wholemount *in situ* hybridization

Tissues were fixed overnight in 4% paraformaldehyde before storing at −20 °C after serial methanol dehydration. The wholemount *in situ* hybridization (WIMSH) protocol as well as the probes used have been previously described (Bogani *et al.*, 2009). A minimum of three samples per genotype were analysed for each probe.

### Chromatin immunoprecipitation

Chromatin immunoprecipitation (ChIP) was performed by modifying published methods of low cell number ChIP (57,58). Briefly, gonads or mesonephroi were fixed in 1% formaldehyde (room temperature, 8min), quenched with 1 volume of 250mM glycine and washed twice with chilled TBSE (20 mM Tris-HCl, 150mM NaCl, 1mM EDTA) before freezing on dry ice and storing at −80 °C. After thawing on ice, 3 pairs of gonads or mesonephroi were pooled and lysed with 100 μL 1% SDS lysis buffer [50mM Tris-HCL pH 8.0, 10mM EDTA, 1% SDS, mini complete inhibitor (Roche)] on ice for 5 min. After centrifugation (500 g, 20 min, 4 °C) samples were resuspended in 100μl dilution buffer (16.7mM Tris-HCl pH 8.0, 167mM NaCl, 1.2mM EDTA, 1.1% Triton X100, 0.01% SDS) and sonicated for 7 cycles (30’ on/30’ off) using a Bioruptor water-bath sonicator (Diagenode, Belgium). After pre-clearing with a mix of protein G and A (Dynabeads, Invitrogen), sonicated chromatin was immunoprecipitated overnight with protein A- and G-coupled antibodies [H3K27Ac (Abcam, Ab4729), 0.5μg; pan-acetylated H3 (MD Millipore, 06–599), 1 μg; H3K9ac (Cell signalling, C5B11), 0.5 μg)]. 10% of each sample (input) was kept and used for calculation of enrichment. On the following day, beads were washed 3 times for 10 min at 4 °C with low salt buffer (20 mM Tris-HCl pH 8.0, 150 mM NaCl, 2mM EDTA, 1% Triton X100, 0.1% SDS). After elution (50 mM Tris-HCl pH 8.0, 10mM EDTA, 1% SDS), samples were digested with proteinase K (2 μg/μl) and reverse cross-linked for 6h at 68 °C. Reverse cross-linked DNA was purified with Agencourt AMPure XP beads and diluted 3-fold before qPCR analysis. qPCR was performed on a 7500 Real-Time PCR system (Applied Biosystems) using Fast SYBR Green Master Mix (Applied Biosystems) according to manufacturer’s instructions. ChIP data were calculated as a ratio to input and were rescaled by normalizing to the control gene *Gapdh*. At least six measurements were made for each histone mark. Statistical differences were determined by using a two-tailed Student’s *t*-test. Primer sequences are available on request.

## Supplementary Material

Supplementary Material is available at *HMG* online.

## Supplementary Material

Supplementary Fig S1

Supplementary Fig S2

Supplementary Fig S3

Supplementary Fig S4

## References

[1] Sekido R., Lovell-Badge R. (2009) Sex determination and SRY: down to a wink and a nudge? Trends Genet., 25, 19–29. 19027189 10.1016/j.tig.2008.10.008

[2] Sekido R., Lovell-Badge R. (2008) Sex determination involves synergistic action of SRY and SF1 on a specific Sox9 enhancer. Nature, 453, 930–934. 10.1038/nature06944 18454134

[3] Carre G.A., Greenfield A. (2014) Characterising novel pathways in testis determination using mouse genetics. Sex. Dev., 8, 199–207. 24643063 10.1159/000358402

[4] Chassot A.A., Ranc F., Gregoire E.P., Roepers-Gajadien H.L., Taketo M.M., Camerino G., de Rooij D.G., Schedl A., Chaboissier M.C. (2008) Activation of beta-catenin signaling by Rspo1 controls differentiation of the mammalian ovary. Hum. Mol. Genet., 17, 1264–1277. 18250098 10.1093/hmg/ddn016

[5] Chassot A.A., Gregoire E.P., Magliano M., Lavery R., Chaboissier M.C. (2008) Genetics of ovarian differentiation: Rspo1, a major player. Sex. Dev., 2, 219–227. 18987496 10.1159/000152038

[6] Jeays-Ward K., Hoyle C., Brennan J., Dandonneau M., Alldus G., Capel B., Swain A. (2003) Endothelial and steroidogenic cell migration are regulated by WNT4 in the developing mammalian gonad. Development, 130, 3663–3670. 12835383 10.1242/dev.00591

[7] Liu C.F., Bingham N., Parker K., Yao H.H. (2009) Sex-specific roles of beta-catenin in mouse gonadal development. Hum. Mol. Genet., 18, 405–417. 18981061 10.1093/hmg/ddn362 PMC2638797

[8] Pannetier M., Chassot A.A., Chaboissier M.C., Pailhoux E. (2016) Involvement of FOXL2 and RSPO1 in Ovarian Determination, Development, and Maintenance in Mammals. Sex. Dev., 10, 167–184. 27649556 10.1159/000448667

[9] Tomizuka K., Horikoshi K., Kitada R., Sugawara Y., Iba Y., Kojima A., Yoshitome A., Yamawaki K., Amagai M., Inoue A. (2008) R-spondin1 plays an essential role in ovarian development through positively regulating Wnt4 signaling. Hum. Mol. Genet., 17, 1278–1291. 18250097 10.1093/hmg/ddn036

[10] Hiramatsu R., Matoba S., Kanai-Azuma M., Tsunekawa N., Katoh-Fukui Y., Kurohmaru M., Morohashi K.I., Wilhelm D., Koopman P., Kanai Y. (2009) A critical time window of Sry action in gonadal sex determination in mice. Development, 136, 129–138. 19036799 10.1242/dev.029587

[11] Pitetti J.L., Calvel P., Romero Y., Conne B., Truong V., Papaioannou M.D., Schaad O., Docquier M., Herrera P.L., Wilhelm D. (2013) Insulin and IGF1 Receptors Are Essential for XX and XY Gonadal Differentiation and Adrenal Development in Mice. PLoS Genet., 9, e1003160. 23300479 10.1371/journal.pgen.1003160 PMC3536656

[12] Bogani D., Siggers P., Brixey R., Warr N., Beddow S., Edwards J., Williams D., Wilhelm D., Koopman P., Flavell R.A. (2009) Loss of mitogen-activated protein kinase kinase kinase 4 (MAP3K4) reveals a requirement for MAPK signalling in mouse sex determination. PLoS Biol., 7, e1000196. 19753101 10.1371/journal.pbio.1000196 PMC2733150

[13] Johnen H., González-Silva L., Carramolino L., Flores J.M., Torres M., Salvador J.M., Herault Y. (2013) Gadd45g is essential for primary sex determination, male fertility and testis development. PLoS One, 8, e58751. 23516551 10.1371/journal.pone.0058751 PMC3596291

[14] Gierl M.S., Gruhn W.H., von Seggern A., Maltry N., Niehrs C. (2012) GADD45G functions in male sex determination by promoting p38 signaling and Sry expression. Dev. Cell, 23, 1032–1042. 23102581 10.1016/j.devcel.2012.09.014

[15] Hammes A., Guo J.K., Lutsch G., Leheste J.R., Landrock D., Ziegler U., Gubler M.C., Schedl A. (2001) Two splice variants of the Wilms' tumor 1 gene have distinct functions during sex determination and nephron formation. Cell, 106, 319–329. 11509181 10.1016/s0092-8674(01)00453-6

[16] Fujimoto Y., Tanaka S.S., Yamaguchi Y.L., Kobayashi H., Kuroki S., Tachibana M., Shinomura M., Kanai Y., Morohashi K., Kawakami K., Nishinakamura R. (2013) Homeoproteins Six1 and Six4 regulate male sex determination and mouse gonadal development. Dev. Cell, 26, 416–430. 23987514 10.1016/j.devcel.2013.06.018

[17] Tevosian S.G., Albrecht K.H., Crispino J.D., Fujiwara Y., Eicher E.M., Orkin S.H. (2002) Gonadal differentiation, sex determination and normal Sry expression in mice require direct interaction between transcription partners GATA4 and FOG2. Development, 129, 4627–4634. 12223418 10.1242/dev.129.19.4627

[18] Warr N, Carre G., Siggers P., Faleato J.V., Brixey R., Pope M., Bogani D., Childers M., Wells S., Scudamore C.L. (2012) Gadd45gamma and Map3k4 interactions regulate mouse testis determination via p38 MAPK-mediated control of Sry expression. Dev. Cell, 23, 1020–1031. 23102580 10.1016/j.devcel.2012.09.016 PMC3526779

[19] Kuroki S., Matoba S., Akiyoshi M., Matsumura Y., Miyachi H., Mise N., Abe K., Ogura A., Wilhelm D., Koopman P. (2013) Epigenetic regulation of mouse sex determination by the histone demethylase Jmjd1a. Science, 341, 1106–1109. 10.1126/science.1239864 24009392

[20] Chan H.M., La Thangue N.B. (2001) p300/CBP proteins: HATs for transcriptional bridges and scaffolds. J. Cell Sci., 114, 2363–2373. 11559745 10.1242/jcs.114.13.2363

[21] Chen S., Seiler J., Santiago-Reichelt M., Felbel K., Grummt I., Voit R. (2013) Repression of RNA polymerase I upon stress is caused by inhibition of RNA-dependent deacetylation of PAF53 by SIRT7. Mol. Cell, 52, 303–313. 24207024 10.1016/j.molcel.2013.10.010

[22] Girdwood D., Bumpass D., Vaughan O.A., Thain A., Anderson L.A., Snowden A.W., Garcia-Wilson E., Perkins N.D., Hay R.T. (2003) P300 transcriptional repression is mediated by SUMO modification. Mol. Cell, 11, 1043–1054. 12718889 10.1016/s1097-2765(03)00141-2

[23] Yao T.P., Oh S.P., Fuchs M., Zhou N.D., Ch'ng L.E., Newsome D., Bronson R.T., Li E., Livingston D.M., Eckner R. (1998) Gene dosage-dependent embryonic development and proliferation defects in mice lacking the transcriptional integrator p300. Cell, 93, 361–372. 9590171 10.1016/s0092-8674(00)81165-4

[24] Tanaka Y., Naruse I., Hongo T., Xu M., Nakahata T., Maekawa T., Ishii S. (2000) Extensive brain hemorrhage and embryonic lethality in a mouse null mutant of CREB-binding protein. Mech. Dev., 95, 133–145. 10906457 10.1016/s0925-4773(00)00360-9

[25] Roelfsema J.H., Peters D.J. (2007) Rubinstein-Taybi syndrome: clinical and molecular overview. Expert. Rev. Mol. Med., 9, 1–16. 10.1017/S1462399407000415 17942008

[26] Liu Y., Wang L., Han R., Beier U.H., Akimova T., Bhatti T., Xiao H., Cole P.A., Brindle P.K., Hancock W.W. (2014) Two histone/protein acetyltransferases, CBP and p300, are indispensable for Foxp3+ T-regulatory cell development and function. Mol. Cell. Biol., 34, 3993–4007. 25154413 10.1128/MCB.00919-14 PMC4386456

[27] Hennig A.K., Peng G.H., Chen S. (2013) Transcription coactivators p300 and CBP are necessary for photoreceptor-specific chromatin organization and gene expression. PLoS One, 8, e69721. 23922782 10.1371/journal.pone.0069721 PMC3724885

[28] Wolf L., Harrison W., Huang J., Xie Q., Xiao N., Sun J., Kong L., Lachke S.A., Kuracha M.R., Govindarajan V. (2013) Histone posttranslational modifications and cell fate determination: lens induction requires the lysine acetyltransferases CBP and p300. Nucleic Acids Res., 41, 10199–10214. 24038357 10.1093/nar/gkt824 PMC3905850

[29] Boussouar F., Goudarzi A., Buchou T., Shiota H., Barral S., Debernardi A., Guardiola P., Brindle P., Martinez G., Arnoult C. (2014) A specific CBP/p300-dependent gene expression programme drives the metabolic remodelling in late stages of spermatogenesis. Andrology, 2, 351–359. 10.1111/j.2047-2927.2014.00184.x 24522976

[30] Valor L.M., Pulopulos M.M., Jimenez-Minchan M., Olivares R., Lutz B., Barco A. (2011) Ablation of CBP in forebrain principal neurons causes modest memory and transcriptional defects and a dramatic reduction of histone acetylation but does not affect cell viability. J. Neurosci., 31, 1652–1663. 21289174 10.1523/JNEUROSCI.4737-10.2011 PMC6623752

[31] Pan Q., Wu Y., Lin T., Yao H., Yang Z., Gao G., Song E., Shen H. (2009) Bone morphogenetic protein-2 induces chromatin remodeling and modification at the proximal promoter of Sox9 gene. Biochem. Biophys. Res. Commun., 379, 356–361. 19103169 10.1016/j.bbrc.2008.12.062

[32] Tsuda M., Takahashi S., Takahashi Y., Asahara H. (2003) Transcriptional co-activators CREB-binding protein and p300 regulate chondrocyte-specific gene expression via association with Sox9. J. Biol. Chem., 278, 27224–27229. 12732631 10.1074/jbc.M303471200

[33] Furumatsu T., Tsuda M., Yoshida K., Taniguchi N., Ito T., Hashimoto M., Ito T., Asahara H. (2005) Sox9 and p300 cooperatively regulate chromatin-mediated transcription. J. Biol. Chem., 280, 35203–35208. 16109717 10.1074/jbc.M502409200

[34] Furumatsu T., Tsuda M., Taniguchi N., Tajima Y., Asahara H. (2005) Smad3 induces chondrogenesis through the activation of SOX9 via CREB-binding protein/p300 recruitment. J. Biol. Chem., 280, 8343–8350. 15623506 10.1074/jbc.M413913200

[35] Buaas F.W., Val P., Swain A. (2009) The transcription co-factor CITED2 functions during sex determination and early gonad development. Hum. Mol. Genet., 18, 2989–3001. 19457926 10.1093/hmg/ddp237

[36] Combes A.N., Spiller C.M., Harley V.R., Sinclair A.H., Dunwoodie S.L., Wilhelm D., Koopman P. (2010) Gonadal defects in Cited2-mutant mice indicate a role for SF1 in both testis and ovary differentiation. Int. J. Dev. Biol., 54, 683–689. 19757380 10.1387/ijdb.092920ac

[37] Benko S., Gordon C.T., Mallet D., Sreenivasan R., Thauvin-Robinet C., Brendehaug A., Thomas S., Bruland O., David M., Nicolino M. (2011) Disruption of a long distance regulatory region upstream of SOX9 in isolated disorders of sex development. J. Med. Genet., 48, 825–830. 22051515 10.1136/jmedgenet-2011-100255

[38] Lybæk H., de Bruijn D., den Engelsman-van Dijk A.H., Vanichkina D., Nepal C., Brendehaug A., Houge G. (2014) RevSex duplication-induced and sex-related differences in the SOX9 regulatory region chromatin landscape in human fibroblasts. Epigenetics, 9, 416–427. 24351654 10.4161/epi.27474 PMC4053460

[39] Hyon C., Chantot-Bastaraud S., Harbuz R., Bhouri R., Perrot N., Peycelon M., Sibony M., Rojo S., Piguel X., Bilan F. (2015) Refining the regulatory region upstream of *SOX9* associated with 46, XX testicular disorders of sex development (DSD). Am. J. Med. Genet. A, 167, 1851–1858. 10.1002/ajmg.a.37101 25900885

[40] Thevenet L., Mejean C., Moniot B., Bonneaud N., Galeotti N., Aldrian-Herrada G., Poulat F., Berta P., Benkirane M., Boizet-Bonhoure B. (2004) Regulation of human SRY subcellular distribution by its acetylation/deacetylation. Embo J., 23, 3336–3345. 15297880 10.1038/sj.emboj.7600352 PMC514523

[41] Zhou B., Ma Q., Rajagopal S., Wu S.M., Domian I., Rivera-Feliciano J., Jiang D., von Gise A., Ikeda S., Chien K.R. (2008) Epicardial progenitors contribute to the cardiomyocyte lineage in the developing heart. Nature, 454, 109–113. 18568026 10.1038/nature07060 PMC2574791

[42] Kreidberg J.A., Sariola H., Loring J.M., Maeda M., Pelletier J., Housman D., Jaenisch R. (1993) WT-1 is required for early kidney development. Cell, 74, 679–691. 8395349 10.1016/0092-8674(93)90515-r

[43] Jameson S.A., Natarajan A., Cool J., DeFalco T., Maatouk D.M., Mork L., Munger S.C., Capel B. (2012) Temporal transcriptional profiling of somatic and germ cells reveals biased lineage priming of sexual fate in the fetal mouse gonad. PLoS Genet., 8, e1002575. 22438826 10.1371/journal.pgen.1002575 PMC3305395

[44] Chen M., Zhang L., Cui X., Lin X., Li Y., Wang Y., Wang Y., Qin Y., Chen D., Han C., (2017) Wt1 directs the lineage specification of sertoli and granulosa cells by repressing Sf1 expression. Development, 144, 44–53. 27888191 10.1242/dev.144105

[45] Bradford S.T., Wilhelm D., Bandiera R., Vidal V., Schedl A., Koopman P. (2009) A cell-autonomous role for WT1 in regulating *Sry* in vivo. Hum. Mol. Genet., 18, 3429–3438. 19549635 10.1093/hmg/ddp283

[46] Warr N., Siggers P., Carre G.A., Wells S., Greenfield A. (2016) Genetic Analyses Reveal Functions for MAP2K3 and MAP2K6 in Mouse Testis Determination. Biol. Reprod., 94, 103. 27009039 10.1095/biolreprod.115.138057 PMC5842889

[47] Bouma G.J., Washburn L.L., Albrecht K.H., Eicher E.M. (2007) Correct dosage of Fog2 and Gata4 transcription factors is critical for fetal testis development in mice. *Proc*. Natl. Acad. Sci. U S A, 104, 14994–14999. 10.1073/pnas.0701677104 PMC1986601 17848526

[48] Warr N., Siggers P., Carre G.A., Bogani D., Brixey R., Akiyoshi M., Tachibana M., Teboul L., Wells S., Sanderson J. (2014) Transgenic expression of *Map3k4* rescues *T*-associated sex reversal (*Tas*) in mice. Hum. Mol. Genet., 23, 3035–3044. 24452333 10.1093/hmg/ddu020 PMC4014197

[49] Wilhelm D., Washburn L.L., Truong V., Fellous M., Eicher E.M., Koopman P. (2009) Antagonism of the testis- and ovary-determining pathways during ovotestis development in mice. Mech. Dev., 126, 324–336. 19269320 10.1016/j.mod.2009.02.006 PMC2680453

[50] Jin Q., Yu L.R., Wang L., Zhang Z., Kasper L.H., Lee J.E., Wang C., Brindle P.K., Dent S.Y., Ge K. (2011) Distinct roles of GCN5/PCAF-mediated H3K9ac and CBP/p300-mediated H3K18/27ac in nuclear receptor transactivation. Embo J., 30, 249–262. 21131905 10.1038/emboj.2010.318 PMC3025463

[51] Larney C., Bailey T.L., Koopman P. (2014) Switching on sex: transcriptional regulation of the testis-determining gene *Sry*. Development, 141, 2195–2205. 10.1242/dev.107052 24866114 PMC4034426

[52] Bedford D.C., Kasper L.H., Fukuyama T., Brindle P.K. (2010) Target gene context influences the transcriptional requirement for the KAT3 family of CBP and p300 histone acetyltransferases. Epigenetics, 5, 9–15. 20110770 10.4161/epi.5.1.10449 PMC2829352

[53] Eggers S., Sadedin S., van den Bergen J.A., Robevska G., Ohnesorg T., Hewitt J., Lambeth L., Bouty A., Knarston I.M., Tan T.Y. (2016) Disorders of sex development: insights from targeted gene sequencing of a large international patient cohort. Genome Biol., 17, 243. 27899157 10.1186/s13059-016-1105-y PMC5126855

[54] Bagheri-Fam S., Ono M., Li L., Zhao L., Ryan J., Lai R., Katsura Y., Rossello F.J., Koopman P., Scherer G. (2015) FGFR2 mutation in 46, XY sex reversal with craniosynostosis. Hum. Mol. Genet., 24, 6699–6710. 26362256 10.1093/hmg/ddv374 PMC4634374

[55] Zhang Z., Hofmann C., Casanova E., Schutz G., Lutz B. (2004) Generation of a conditional allele of the CBP gene in mouse. Genesis, 40, 82–89. 15452871 10.1002/gene.20068

[56] Kasper L.H., Fukuyama T., Biesen M.A., Boussouar F., Tong C., de Pauw A., Murray P.J., van Deursen J.M., Brindle P.K. (2006) Conditional knockout mice reveal distinct functions for the global transcriptional coactivators CBP and p300 in T-cell development. Mol. Cell Biol., 26, 789–809. 16428436 10.1128/MCB.26.3.789-809.2006 PMC1347027

[57] Ng J.H., Kumar V., Muratani M., Kraus P., Yeo J.C., Yaw L.P., Xue K., Lufkin T., Prabhakar S., Ng H.H. (2013) In Vivo Epigenomic Profiling of Germ Cells Reveals Germ Cell Molecular Signatures. Dev. Cell, 24, 324–333. 23352811 10.1016/j.devcel.2012.12.011

[58] Zylicz J.J., Dietmann S., Gunesdogan U., Hackett J.A., Cougot D., Lee C., Surani M.A. (2015) Chromatin dynamics and the role of G9a in gene regulation and enhancer silencing during early mouse development. eLife, 4. 10.7554/eLife.09571 PMC4729692 26551560

