# Supplementary figures and images for: Loss of p300 and CBP disrupts histone acetylation at the mouse Sry promoter and causes XY gonadal sex reversal
Source: Hum Mol Genet. 2017 Nov 14;27(1):190–8. doi: 10.1093/hmg/ddx398 (PMC5886154; doi:10.1093/hmg/ddx398)

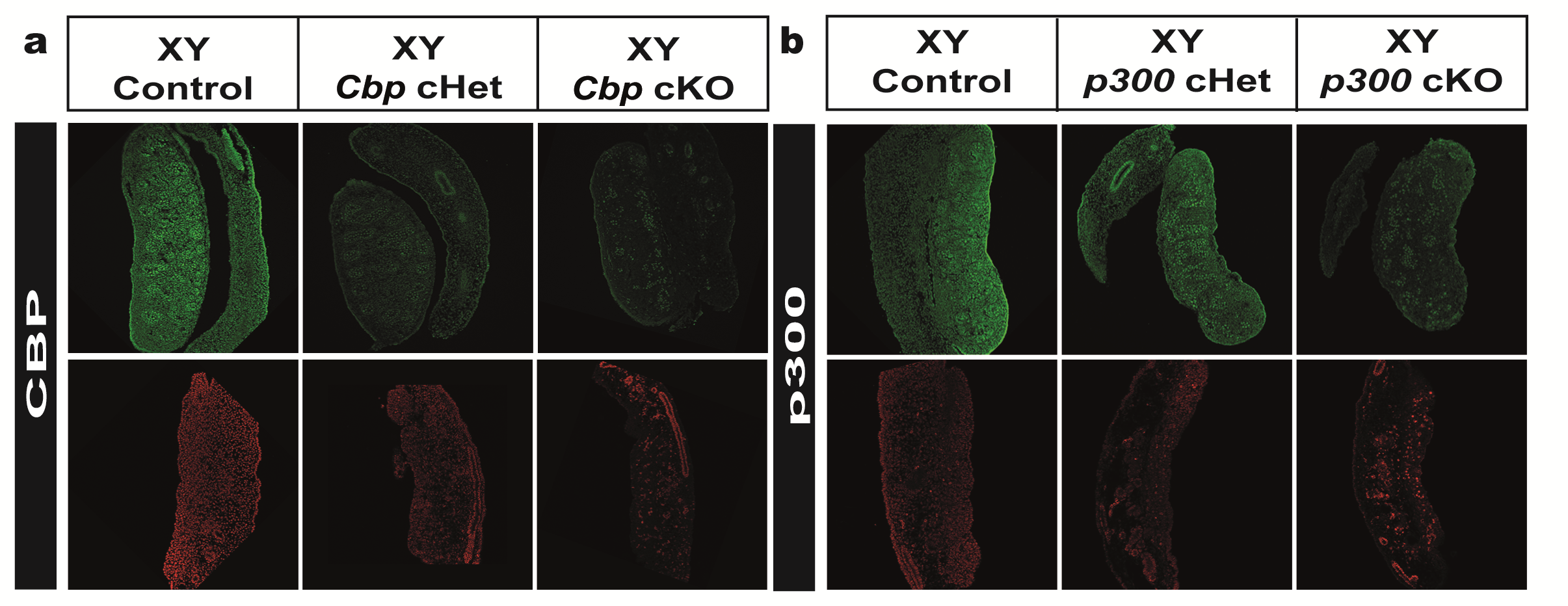

Supplement: Supplementary Fig S1 [file ddx398_fig_s1.png]

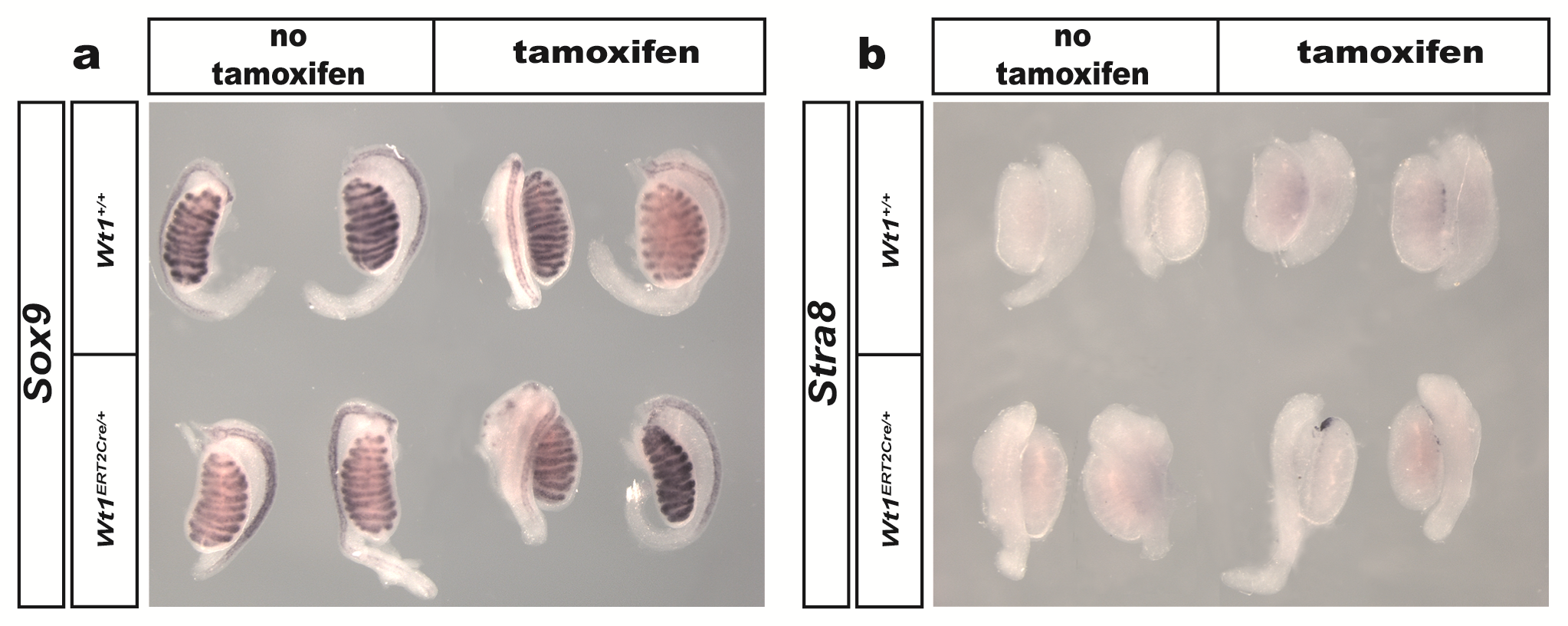

Supplement: Supplementary Fig S2 [file ddx398_fig_s2.png]

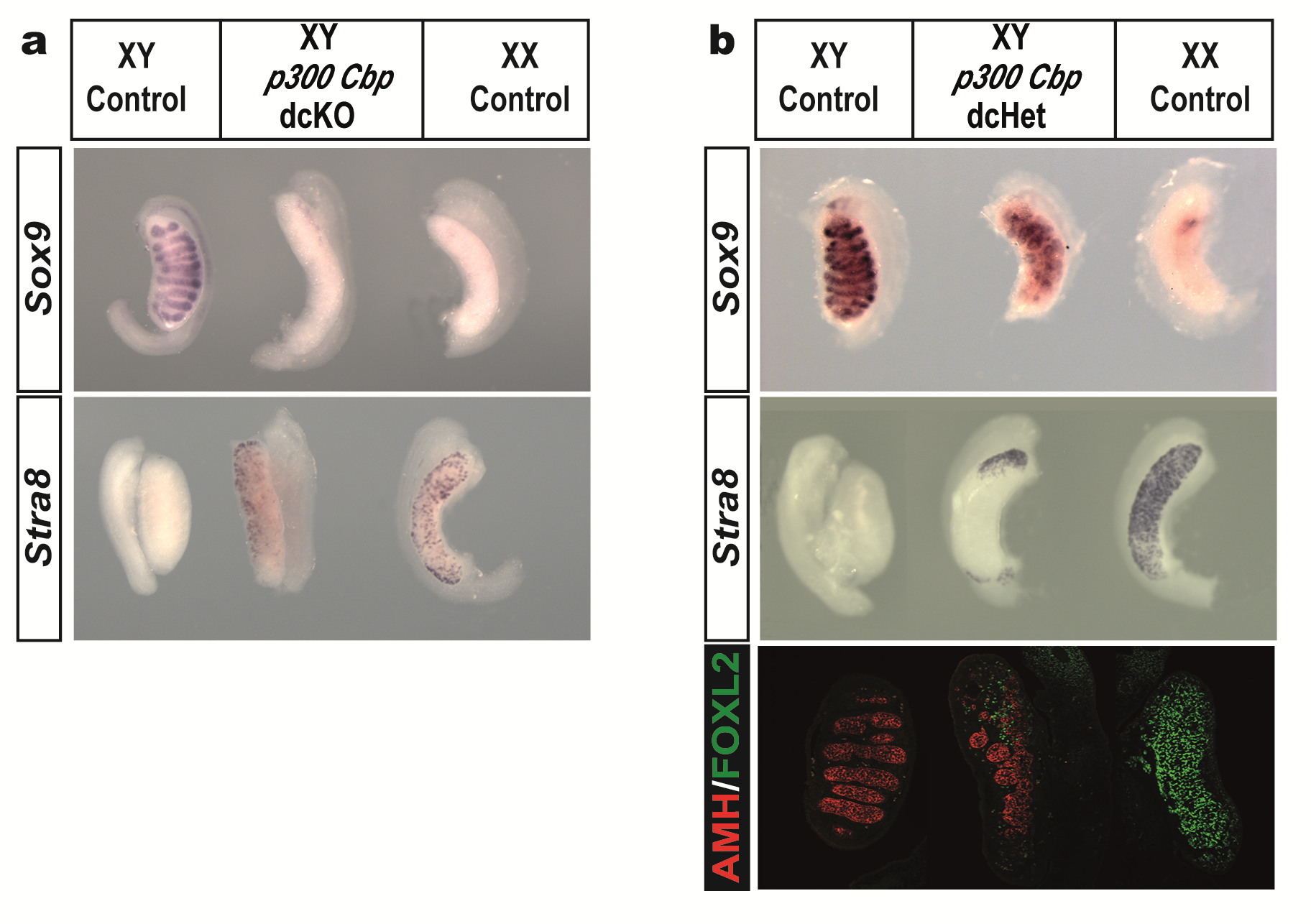

Supplement: Supplementary Fig S3 [file ddx398_fig_s3.png]

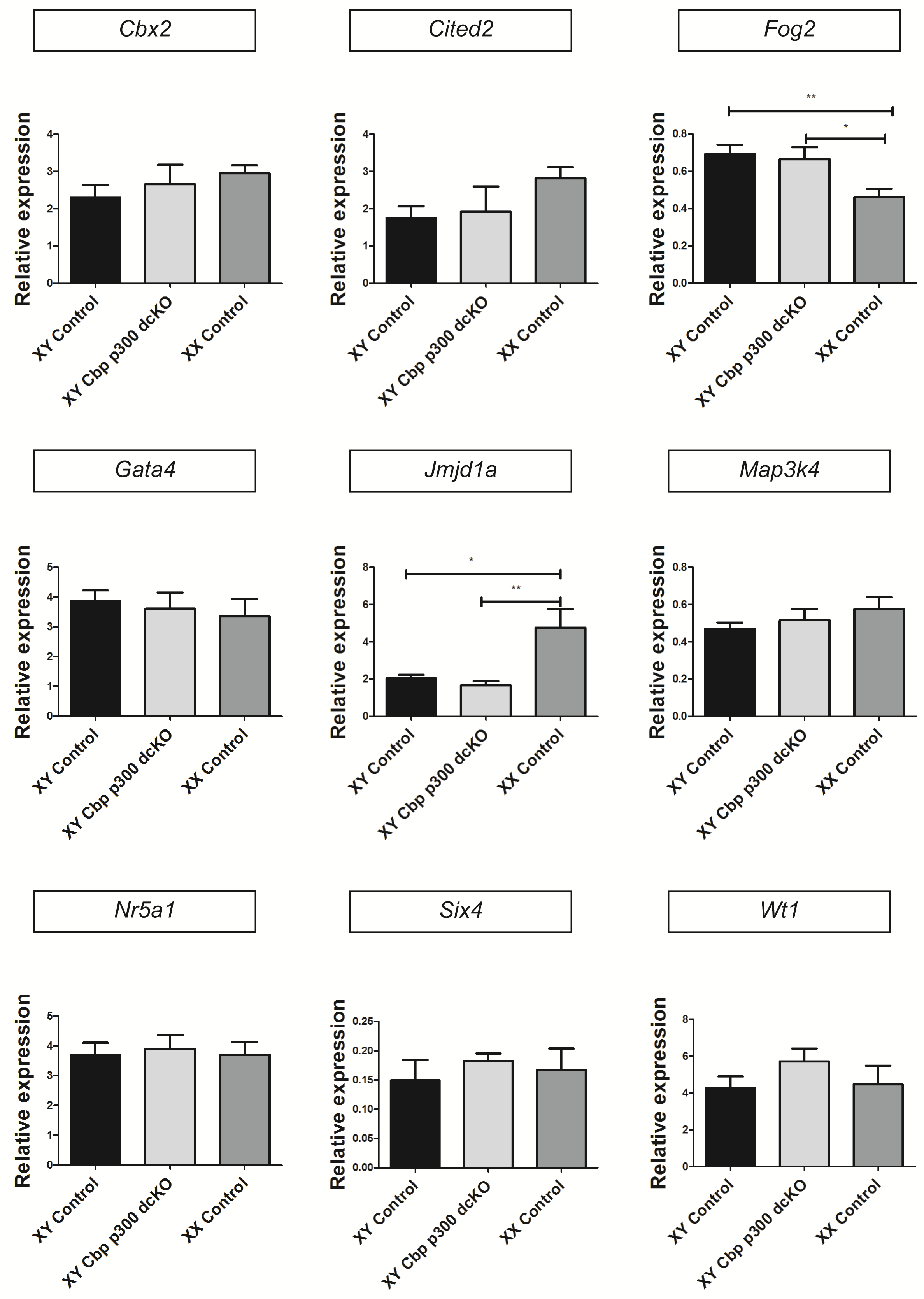

Supplement: Supplementary Fig S4 [file ddx398_fig_s4.png]
